# Determinação dos Níveis Séricos de Glicogênio Sintase 3 Beta em Pacientes com Insuficiência Cardíaca, um Novo Marcador para Diagnóstico e Definição da Gravidade da Doença?

**DOI:** 10.36660/abc.20240155

**Published:** 2024-10-30

**Authors:** Gokhan Altunbas, Mehmet Kaplan, Veysel Duzen, Emin Erdem Kaya, Hafize Gokce Gokdeniz, Seyithan Taysi

**Affiliations:** 1 Department of Cardiology Gaziantep University School of Medicine Gaziantep Turquia Department of Cardiology, Gaziantep University School of Medicine, Gaziantep – Turquia; 2 Gaziantep City Hospital Training and Research Hospital Gaziantep Turquia Gaziantep City Hospital, Training and Research Hospital, Gaziantep – Turquia; 3 Hatay Dortyol State Hospital Hatay Turquia Hatay Dortyol State Hospital, Hatay – Turquia; 4 Department of Biochemistry Gaziantep University School of Medicine Gaziantep Turquia Department of Biochemistry, Gaziantep University School of Medicine, Gaziantep – Turquia

**Keywords:** Quinase 3 da Glicogênio Sintase, Insuficiência Cardíaca, Peptídeos Natriuréticos

## Abstract

**Fundamento:**

A glicogênio sintase quinase 3β (GSK3β) é uma enzima que tem papéis na patogênese da insuficiência cardíaca (IC). Tentamos revelar os níveis séricos de GSK3β em tipos de IC.

**Objetivos:**

Neste estudo, avaliamos os níveis séricos de GSK3β em pacientes com IC. Além disso, tentamos elucidar qualquer possível relação entre os níveis séricos de GSK3β e a gravidade da doença entre três tipos diferentes de pacientes com IC.

**Métodos:**

Realizamos um estudo prospectivo e inscrevemos 112 pacientes: 50 pacientes no grupo IC com fração de ejeção preservada (ICFEp), 30 pacientes no grupo IC com FE levemente reduzida (ICFEmr) e 32 pacientes no grupo IC com FE reduzida (ICFEr). Também avaliamos 50 controles saudáveis. Exames ecocardiográficos foram realizados. Medimos a GSK-3β sérica e o peptídeo natriurético tipo B N-terminal (NT-proBNP). Medimos os níveis de proteína C-reativa altamente sensível (PCR-as) e calculamos a razão neutrófilo-linfócito (NLR) e a razão plaquetas-linfócitos (PLR) a partir da contagem do hemograma. A significância estatística aceita foi p < 0,05.

**Resultados:**

Os níveis séricos de GSK3β foram significativamente maiores entre pacientes com IC em comparação com controles saudáveis (níveis medianos de GSK3β; 117,26 (45,39 -223,85) vs 13,91 (5,6 -23,3) ng/mL, p < 0,001). Além disso, os níveis de GSK3β foram maiores entre pacientes com ICFEp e menores entre pacientes com ICFEr; 236,44 (132,89 -432) vs. 38,72 (23,15-67,31) ng/mL, respectivamente (p < 0,001). Os níveis medianos de NT-proBNP, como esperado, foram significativamente maiores entre pacientes com IC em comparação com controles saudáveis (660 (291 -1000) vs. 92 (78 -102) pg/mL, p<0,001). Como um marcador de inflamação sistêmica, os valores de hsCRP, NLR e PLR não diferiram significativamente entre pacientes com IC e controles. Conclusão: Os níveis de GSK3β foram significativamente maiores entre pacientes com IC. Além disso, à medida que a fração de ejeção diminui, os níveis de GSK3β também se reduzem, provavelmente como um mecanismo de proteção para evitar mais apoptose e morte de miócitos.

## Graphical abstract

**Figura Central:**
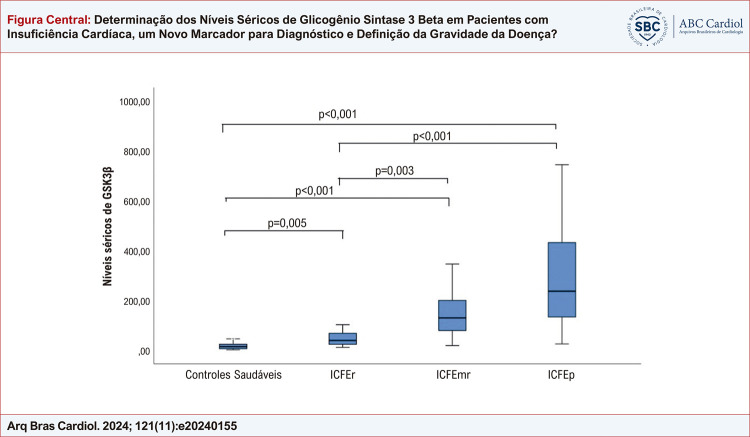
: Determinação dos Níveis Séricos de Glicogênio Sintase 3 Beta em Pacientes com Insuficiência Cardíaca, um Novo Marcador para Diagnóstico e Definição da Gravidade da Doença?

## Introdução

A incidência e prevalência de insuficiência cardíaca (IC) estão aumentando em todo o mundo. Devido aos avanços cada vez maiores no tratamento de doença arterial coronária, doença cardíaca valvular, arritmias e até mesmo cardiomiopatias inflamatórias e genéticas, a probabilidade de sobrevivência aumenta, e isso resulta em mais pacientes vivendo com IC. Além disso, o envelhecimento da população e o aumento da incidência de hipertensão levam a um aumento no número de pacientes com insuficiência cardíaca com fração de ejeção preservada (ICFEp).^1^ Com base nas diretrizes recentes da Sociedade Europeia de Cardiologia sobre a definição de insuficiência cardíaca, existem três tipos de insuficiência cardíaca com base na fração de ejeção do ventrículo esquerdo (FEVE): insuficiência cardíaca com fração de ejeção reduzida (ICFEr), onde a FEVE é ≤40%, insuficiência cardíaca com fração de ejeção levemente reduzida (ICFEmr), onde a FE está entre 41 e 49% e, finalmente; ICFEp, onde a FE é ≥50%.^2^

A glicogênio sintase quinase 3 (GSK 3β) é uma serina-treonina quinase que foi descoberta primeiro como a principal enzima responsável pelo metabolismo do glicogênio. No entanto, descobertas recentes sugerem que essa enzima tem vários papéis em uma infinidade de funções celulares, incluindo regulação de fatores de transcrição, embriogênese, progressão do ciclo celular, proliferação celular, fibrose, apoptose, hipertrofia miocárdica e até mesmo expressão gênica. No que diz respeito, há vários estudos indicando seu papel em vários estados de doença, incluindo lesão de isquemia-reperfusão, câncer, doença de Alzheimer e acidente vascular cerebral.^3-5^ Quanto à doença cardiovascular, a principal área de interesse é o papel da GSK 3 na lesão de isquemia-reperfusão. Recentemente, o papel da GSK 3 na IC ganhou mais interesse. As principais descobertas sugerem que a inibição sustentada da GSK3 induz hipertrofia e inibe a apoptose, e essa regulação negativa na IC pode ser vista como uma resposta compensatória e mecanismo de proteção.^6^ À luz desta pesquisa, queríamos avaliar os níveis séricos de GSK-3 beta em três perfis diferentes de IC e comparar os resultados com indivíduos saudáveis.

## Métodos

Neste estudo, inscrevemos pacientes com IC e controles saudáveis entre 18 e 65 anos de idade. Com base nas diretrizes mais recentes da Sociedade Europeia de Cardiologia sobre insuficiência cardíaca,^2^ categorizamos os pacientes com insuficiência cardíaca em três grupos: 50 pacientes no grupo de ICFEp, 30 pacientes no grupo de ICFEmr e 32 pacientes no grupo de ICFEr. Também incluímos 50 controles saudáveis para comparar os resultados com pacientes com IC. Determinamos o número de pacientes em cada grupo por conveniência. A GSK-3β (BT Lab, Número de catálogo: E3196Hu, China) foi determinada com um kit comercial. A intensidade da cor gerada pelo método de ensaio imunoenzimático (ELISA) foi medida com um leitor de ELISA (Biotek ELx800, EUA). Todos os procedimentos foram realizados na Faculdade de Medicina da Universidade de Gaziantep, Departamento de Bioquímica. Os resultados são expressos como ng/mL. O nível de pró-peptídeo natriurético tipo B N-terminal (NT-proBNP) foi medido com um analisador de hemoglobina totalmente automático e dispositivos UNICELL-S (Shenzen, China). Os resultados são expressos como pg/mL. Os níveis de proteína C-reativa alta sensibilidade (PCR-as) sérica foram determinados com o dispositivo Beckman Coulter Chemistry Analyzer AU5800 (Beckman Coulter Inc., Brea, CA 92821 EUA). Os resultados são expressos como mg/mL.

### Avaliação ecocardiográfica

As medidas ecocardiográficas transtorácicas foram registradas com base nos padrões da Sociedade Americana de Ecocardiografia. A FEVE foi determinada usando a fórmula 2D de Simpson nas vistas apicais de quatro câmaras e duas câmaras. Para determinar a presença de disfunção diastólica para ICFEp, usamos três determinantes principais da disfunção diastólica: velocidades diastólicas precoces (E) e tardias (A) do ventrículo esquerdo que foram registradas nas pontas dos folhetos da valva mitral na vista apical de quatro câmaras, velocidades diastólicas Doppler tecidual (e’) registradas no septo interventricular e na parede livre lateral e velocidade do jato de regurgitação da valva tricúspide.^7^ Além dessas variáveis ecocardiográficas, sintomas clínicos e achados de IC também foram necessários para o diagnóstico de ICFEp.

### Análise estatística

Variáveis contínuas com distribuição normal foram descritas por meio de média ± desvio padrão, e variáveis contínuas sem distribuição normal foram descritas por meio de mediana e intervalo interquartil. Para variáveis categóricas, os dados foram expressos como frequência e porcentagem. Os parâmetros bioquímicos foram avaliados com o teste de Shapiro-Wilk e revelaram que não apresentaram distribuição normal (p<0,05). Ao comparar essas variáveis aos grupos de estudo, foram usados os test U de Mann-Whitney ou o de Kruskal Wallis. Para determinar a diferença entre os grupos, foi usado o teste de comparação múltipla de Dunn. Além disso, a análise de correlação de Spearman foi usada para avaliar a associação entre variáveis numéricas. As análises foram realizadas usando o SPSS 22.0, e p < 0,05 foi determinado como o nível de significância estatística.

## Resultados

Inscrevemos 112 pacientes com IC e 50 controles saudáveis. A idade média da população total do estudo foi de 59,57 ± 9,24 anos. As características basais dos pacientes e controles foram resumidas na Tabela 1. A idade média foi semelhante entre os grupos de pacientes e controles e entre pacientes com diferentes grupos de IC. Como um marcador de gravidade da doença e prognóstico, o marcador característico da IC, os níveis medianos do peptídeo natriurético tipo B N-terminal (NT-proBNP) foram significativamente maiores no grupo de pacientes em comparação aos controles saudáveis (660 (291 -1000) vs. 92 (78 -102) p < 0,001). Além disso, houve um aumento significativo nos níveis de NT-proBNP com a diminuição da fração de ejeção (p < 0,01). Os níveis séricos de GSK-3β foram significativamente aumentados em pacientes com IC em comparação ao controle saudável (117,26 (45,39 -223,85) vs 13,91 (5,6 -23,3) p < 0,001). Os pacientes com IC foram divididos em três grupos com base na FEVE: a) ICFEp onde a FEVE é ≥ 50%, b) ICFEmr onde a FEVE é ≥ 40%, mas < 50% e c) ICFEr onde a FEVE é < 40%. Ao considerar diferentes grupos de IC com base na FEVE, os níveis de GSK-3β foram mais baixos entre os pacientes com ICFEr (mediana 38,72) e mais altos entre os pacientes com ICFEp (mediana 236,44) (p < 0,001). Ao avaliar pacientes com IC, em três grupos com base na FEVE, os níveis de GSK-3β foram significativamente menores entre os pacientes com frações de ejeção do ventrículo esquerdo mais baixas. Entre os pacientes com ICFEP, os níveis medianos de GSK-3β foram 236,44, enquanto os níveis séricos de GSK-3β foram 129,2 para pacientes com ICFEM e 38,72 para pacientes com ICFEr (p<0,001). Os níveis séricos de BNP também aumentaram significativamente com a diminuição das frações de ejeção (p<0,001). Como um marcador de inflamação e prognóstico, os níveis de PCR-as e as razões NLR foram semelhantes entre os diferentes grupos de IC com base na FEVE. As diferenças e comparações dentro do grupo são resumidas na Figura Central e na Tabela 2.

**Tabela 1:** – Características basais dos pacientes e controles

	Controles	Pacientes com insuficiência cardíaca	p
	Mediana (Q1-Q3)	Mediana (Q1-Q3)	
GSK-3β (ng/mL)	13,91 (5,6 -23,3)	117,26 (45,39 -223,85)	0,001***
NT-proBNP (pg/mL)	92 (78 -102 )	660 (291 -1000 )	0,001***
FEVE (%)	55 (55 -57 )	44,5 (35 -53,5 )	0,001***
PCR-as (mg/L)	5 (2 -8,4)	4,78 (2 -7,05 )	0,405
Leucócito	9 (7,21 -11,53)	9,71 (7,21 -11,53)	0,878
Plaquetas	258,5 (225 -311 )	281 (237 -333 )	0,252
Linfócito	2,23 (1,78 -4,87)	2,31 (1,5 -3,58)	0,187
Neutrófilo	7,1 (4,96 -7,77 )	6,01 (4,44 -7,77)	0,330
Creatinina	0,87 (0,71 -1,01)	0,77 (0,7 -0,99)	0,148
TFGe	82,36 (65 -109 )	99,5 (72 -109,5 )	0,200
NLR	2,06 (1,12 -4,02)	2,32 (1,61 -4,01)	0,212
PLR	111,05 (57,7 -150,25)	123,93 (79,21 -180,99)	0,069

***p<0,001; Teste U de Mann-Whitney. GSK-3β: glicogênio sintase quinase 3 beta; NT-proBNP: peptídeo natriurético cerebral N-terminal; FEVE: fração de ejeção do ventrículo esquerdo; PCR-as: proteína C reativa altamente sensível; TFGe: taxa de filtração glomerular estimada (mL/min/1,73 m^2^); NLR: razão neutrófilos/linfócitos; PLR: razão plaquetas/linfócitos.

**Tabela 2:** – Diferenças e comparações dentro do grupo

Variáveis	Controles saudáveis e tipos de insuficiência cardíaca	Mediana (Q1-Q3)	p
GSK-3β (ng/mL)	Controles saudáveis	13,91 (5,6 -23,3)	0,001***
	ICFEr	38,72 (23,15 -67,31)	
	ICFEmr	129,2 (78,9 -199,63)	
	ICFEp	236,44 (132,89 -432)	
NT-proBNP (pg/mL)	Controles saudáveis	92 (78 -102)	0,001***
	ICFEr	1110 (817,5 -2220)	
	ICFEmr	607 (430 -780)	
	ICFEp	225 (180 -312)	
PCR-as (mg/L)	Controles saudáveis	5 (2 -8,4)	0,425
	ICFEr	5 (2,44 -9)	
	ICFEmr	5 (1 -6)	
	ICFEp	3,64 (2 -6)	
Leucócito	Controles saudáveis	9 (7,21 -11,53)	0,998
	ICFEr	9,86 (7,12 -11,77)	
	ICFEmr	9,87 (7,21 -11,06)	
	ICFEp	8,9 (7,67 -12)	
Plaquetas	Controles saudáveis	258,5 (225 -311)	0,550
	ICFEr	272 (237,5 -358,5)	
	ICFEmr	280,5 (228 -321)	
	ICFEp	281 (238 -365)	
Linfócito	Controles saudáveis	2,23 (1,78 -4,87)	0,344
	ICFEr	2,31 (1,53 -4,87)	
	ICFEmr	2,31 (1,56 -2,75)	
	ICFEp	2,34 (1,42 -3,12)	
Neutrófilo	Controles saudáveis	7,1 (4,96 -7,77)	0,025*
	ICFEr	7,59 (5,41 -8,33)	
	ICFEmr	5,95 (4,35 -6,87)	
	ICFEp	4,99 (4,37 -7,77)	
NLR	Controles saudáveis	2,06 (1,12 -4,02)	0,601
	ICFEr	2,13 (1,12 -4,62)	
	ICFEmr	2,62 (1,67 -3,95)	
	ICFEp	2,28 (1,87 -3,49)	
PLR	Controles saudáveis	111,05 (57,7 -150,25)	0,116
	ICFEr	116,22 (53,62 -175,07)	
	ICFEmr	119,25 (86,34 -161,04)	
	ICFEp	147,33 (101,08 -190)	

***: p<0,001 (diferença estatisticamente significativa muito forte). *: p<0,05 (diferença estatisticamente significativa). GSK-3β: glicogênio sintase quinase 3 beta; NT-proBNP: peptídeo natriurético cerebral N-terminal; FEVE: fração de ejeção do ventrículo esquerdo; PCR-as: proteína C reativa altamente sensível; TFGe: taxa de filtração glomerular estimada (mL/min/1,73m^2^); NLR: razão neutrófilos/linfócitos; PLR: razão plaquetas/linfócitos.

## Discussão

Neste estudo, avaliamos marcadores estabelecidos para prognóstico entre pacientes com IC e, como um marcador novo e promissor, medimos os níveis séricos de GSK-3β e comparamos os resultados com controles saudáveis. A principal descoberta do estudo foi que os níveis séricos de GSK-3β aumentaram significativamente entre pacientes com IC. Além disso, com a redução da fração de ejeção, os níveis de GSK-3β tiveram uma tendência à redução. Os níveis mais altos de níveis de GSK-3β foram registrados entre pacientes com ICFEp. A glicogênio sintase quinase (GSK) tem duas isoformas, α e β. Para mecanismos específicos do coração, a isoforma β ganhou muito interesse. Estudos recentes revelaram que a GSK-3β tem um papel importante no pré-condicionamento isquêmico, pós-condicionamento, hipertrofia e IC. Entre várias vias e mecanismos, o efeito da GSK-3β na fisiologia cardíaca é exercido pela fosforilação de reguladores transcricionais e fatores de iniciação translacional.^8^ A GSK-3β ativada previne o crescimento hipertrófico de miócitos e, especialmente no processo de avanço da IC, a GSK-3β é inibida provavelmente como um mecanismo de proteção.^9^ Em nosso estudo, os níveis séricos de GSK-3β foram mais altos entre pacientes com ICFEp. Com a redução das frações de ejeção, os níveis de GSK-3β estavam diminuindo constantemente, e essa diferença atingiu uma diferença fortemente significativa (p<0,001). Nossas descobertas apoiam descobertas de pesquisas anteriores de que, com o avanço da IC, a GSK-3 é regulada negativamente como um mecanismo de proteção. Outro suporte para essa descoberta vem de um estudo animal. Kirk et al. avaliaram corações de cães com IC com dissincronia.^1^º Eles usaram terapia de ressincronização cardíaca (TRC) para esses animais e descobriram que o GSK-3β foi desativado nesses corações e reativado pela TRC. Além disso, a TRC melhorou a responsividade ao cálcio dos miofilamentos por meio da reativação do GSK-3. Essa descoberta também sugere uma possível abordagem terapêutica para terapias de IC em um futuro próximo. Nós medimos os níveis séricos de BNP, que é aceito como o biomarcador padrão para IC tanto no diagnóstico quanto na determinação do prognóstico e também na avaliação da resposta ao tratamento.^2^ Em comparação com controles saudáveis, os níveis medianos de NT-proBNP foram significativamente maiores entre pacientes com IC. Essa associação foi positivamente correlacionada com os níveis de GSK-3β, que também foram significativamente aumentados entre pacientes com IC em comparação com controles saudáveis. A partir deste ponto, podemos assumir que o GSK-3β pode ser usado como um novo biomarcador para IC como o BNP. No entanto, estudos adicionais com um número maior de pacientes são necessários para esclarecer essa associação.

Como um fator de risco estabelecido para eventos cardiovasculares e especialmente coronários futuros, também medimos os níveis séricos de PCR-as. Tanto para doença arterial coronária quanto para IC, a PCR-as é um marcador estabelecido para prognóstico. Os níveis séricos de PCR-as foram semelhantes entre controles saudáveis e pacientes com IC. Outros marcadores amplamente estudados de inflamação sistêmica são a razão neutrófilos-linfócitos (NLR) e a razão plaquetas-linfócitos (PLR). A inflamação sistêmica é aceita como uma etapa patogênica fundamental tanto para o desenvolvimento de aterosclerose quanto para IC. Além disso, um nível aumentado de inflamação sistêmica é aceito como um marcador de prognóstico ruim para eventos coronários agudos e IC crônica. Em sua grande metanálise, Dong et al. avaliaram 9.406 pacientes de oito estudos e revelaram que a NLR é um marcador de prognóstico ruim para pacientes com síndrome coronária aguda recente, e valores mais altos de NLR basal foram associados ao aumento da mortalidade hospitalar.^11^ Uthamalingam et al. avaliaram pacientes hospitalizados por IC aguda descompensada e revelaram que valores aumentados de NLR foram independentemente associados à mortalidade a longo prazo.^12^ Em nosso estudo, os valores medianos de NLR foram semelhantes tanto para controles saudáveis e para pacientes com IC (p: 0,212). Além disso, os valores medianos de NLR foram semelhantes entre diferentes subgrupos de IC (p: 0,601). O número relativamente baixo de pacientes pode explicar essa falta de diferença. Além disso, não inscrevemos pacientes com descompensação aguda. Como NLR é geralmente um marcador de inflamação aguda e, portanto, a descompensação aguda de IC provavelmente causaria elevação dos valores de NLR calculados.

### Limitações do estudo e perspectivas futuras

Uma limitação do nosso estudo foi o número relativamente pequeno de pacientes inscritos. O maior número de pacientes produziria dados clinicamente mais relevantes e robustos. A via da glicogênio sintase quinase beta está ganhando interesse crescente na via da IC. Com uma maior compreensão dessa via, novas terapias para IC visando a via GSK podem se tornar disponíveis em um futuro próximo.

## Conclusão

Os níveis séricos de GSK-3β aumentaram significativamente entre pacientes com IC em comparação com controles saudáveis. Além disso, os níveis de GSK-3 mostraram uma redução significativa com fração de ejeção reduzida, apontando para um possível mecanismo de proteção e também um alvo terapêutico para o tratamento de IC.
