# Supplementary material for: Determinação dos Níveis Séricos de Glicogênio Sintase 3 Beta em Pacientes com Insuficiência Cardíaca, um Novo Marcador para Diagnóstico e Definição da Gravidade da Doença?
Source: Arq Bras Cardiol. 2024 Oct 30;121(11):e20240155. [Article in Portuguese] doi: 10.36660/abc.20240155 (PMC11634289; doi:10.36660/abc.20240155)
Supplement: Translation

# Translation: Determination of Serum Glycogen Synthase 3 Beta Levels in Patients with Heart Failure, a Novel Marker for Diagnosis and Defining Disease Severity?

**DOI:** 10.36660/abc.20240155i

**Authors:** Gokhan Altunbas, Mehmet Kaplan, Veysel Duzen, Emin Erdem Kaya, Hafize Gokce Gokdeniz, Seyithan Taysi

**Affiliations:** 1 Turkey Department of Cardiology, Gaziantep University School of Medicine, Gaziantep – Turkey; 2 Turkey Gaziantep City Hospital, Training and Research Hospital, Gaziantep –Turkey; 3 Turkey Hatay Dortyol State Hospital, Hatay – Turkey; 4 Turkey Department of Biochemistry, Gaziantep University School of Medicine, Gaziantep – Turkey

**Keywords:** Glycogen Synthase Kinase 3, Heart Failure, Natriuretic Peptides

## Abstract

**Background:**

Glycogen synthase kinase 3β (GSK3β) is an enzyme that has roles in the pathogenesis of heart failure (HF). We try to reveal serum GSK3β levels in types of HF.

**Objectives:**

In this study, we evaluated serum GSK3β levels in HF patients. Also, we tried to elucidate any possible relationship between serum GSK3β levels and disease severity among three different types of HF patients.

**Methods:**

We performed a prospective study and enrolled 112 patients: 50 patients in heart failure with preserved ejection fraction (HFpEF) group, 30 patients in heart failure with mildly reduced ejection fraction (HFmrEF) group, and 32 patients in heart failure with reduced ejection fraction group (HFrEF). We also evaluated 50 healthy controls. Echocardiographic examinations were performed. We measured serum GSK-3β and N-terminal pro-B-type natriuretic peptide (NT-proBNP). We measured highly sensitive C-reactive protein (hs-CRP) levels and calculated neutrophil-lymphocyte ratio (NLR) platelets-to-lymphocyte ratio (PLR) from the hemogram count. Statistical significance was accepted p < 0.05.

**Results:**

Serum GSK3β levels were significantly higher among patients with HF compared to healthy controls (median GSK3β levels; 117.26 (45.39 -223.85) vs 13.91 (5.6 -23.3) ng/mL, p<0.001). Also, GSK3β levels were highest among patients with HFpEF and lowest among patients with HFrEF; 236.44 (132.89 -432) vs. 38.72 (23.15-67.31) ng/mL respectively (p<0.001). Median NT-proBNP levels, as expected, were significantly higher among patients with HF compared to healthy controls (660 (291 -1000) vs. 92 (78 -102) pg/mL, p<0.001). As a marker of systemic inflammation, hsCRP values, NLR, and PLR did not differ significantly among HF patients and controls.

**Conclusion:**

GSK3β levels were significantly higher among patients with HF. Also, as the ejection fraction declines, GSK3β levels also reduce, probably as a protective mechanism to prevent further apoptosis and myocyte death.

## Introduction

The incidence and prevalence of heart failure (HF) is increasing worldwide. Due to ever-growing advances in the treatment of coronary artery disease, valvular heart disease, arrhythmias, and even inflammatory and genetic cardiomyopathies, the probability of survival increases, and this results in more patients living with HF. Also, the aging population and the increased incidence of hypertension lead to an increased number of patients with heart failure with preserved ejection fraction (HFpEF).^1^ Based on the European Society of Cardiology’s recent guidelines on HF definition, there are three types of HF based on left ventricle ejection fraction (LVEF): heart failure with reduced ejection fraction (HFrEF) where LVEF is ≤ 40%, heart failure with mildly reduced ejection fraction (HFmrEF) where EF is between 41 and 49% and finally; HFpEF where EF is ≥ 50%.^2^

Glycogen synthase kinase 3 (GSK 3) is a serine-threonine kinase that was primarily discovered as the main enzyme responsible for glycogen metabolism. However, recent findings suggest that this enzyme has various roles in a multitude of cellular functions, including regulation of transcription factors, embryogenesis, cell cycle progression, cell proliferation, fibrosis, apoptosis, myocardial hypertrophy, and even gene expression. As regards there are numerous studies indicating its role in various disease states, including ischemia-reperfusion injury, cancer, Alzheimer’s disease, and stroke.^3-5^ As for cardiovascular disease, the main area of interest is the role of GSK 3 in ischemia-reperfusion injury. Recently, the role of GSK 3 in HF has gained more interest. The main findings suggest that sustained inhibition of GSK3 induces hypertrophy and inhibits apoptosis, and this downregulation in HF can be viewed as a compensatory response and protective mechanism.^6^ In light of this research, we wanted to evaluate serum levels of GSK-3 beta levels in three different HF profiles and compare the results with healthy subjects.

## Methods

In this study, we enrolled HF patients and healthy controls between 18 and 65 years of age. Based on the most recent European Society of Cardiology guidelines on HF,^2^ we categorized HF patients into three groups: 50 patients in HFpEF group, 30 patients in HFmrEF group and 32 patients in HFrEF. We also included 50 healthy controls to compare the results with HF patients. We determined the number of patients in each group by convenience. The glycogen synthase kinase 3 beta (GSK-3β) (BT Lab, Catalog No: E3196Hu, China) level was determined with a commercial kit. The color intensity generated by the enzyme-linked immunosorbent assay (ELISA) method was measured with an ELISA reader (Biotek ELx800, USA). All procedures were performed at Gaziantep University Faculty of Medicine, Department of Biochemistry. Results are expressed as ng/mL. N-terminal pro-B-type natriuretic peptide (NT-proBNP) level was measured with a fully automatic Hemoglobin Analyzer and UNICELL-S (Shenzen, China) devices. Results are expressed as pg/mL. Serum highly sensitive C-reactive protein (hs-CRP) levels were determined with the Beckman Coulter Chemistry Analyzer AU5800 (Beckman Coulter Inc., Brea, CA 92821 USA) device. Results are expressed as mg/mL.

### Echocardiographic evaluation

Transthoracic echocardiographic measurements were recorded based on American Society of Echocardiography standards. The left ventricular ejection fraction was determined using the 2D Simpson formula from both apical four-chamber and two-chamber views. To determine the presence of diastolic dysfunction for HFpEF group, we used three main determinants of diastolic dysfunction: left ventricle early (E) and late (A) diastolic velocities which were recorded at mitral valve leaflet tips from apical 4-chamber view, tissue Doppler diastolic velocities (e’) recorded at interventricular septum and lateral free wall and tricuspid valve regurgitation jet velocity.^7^ In addition to these echocardiographic variables, clinical symptoms and findings of HF were also needed for the diagnosis of HFpEF.

### Statistical analysis

Continuous variables with normal distribution were described through mean ± standard deviation, and continuous variables without normal distribution were described through median and interquartile range. For categorical variables, data were expressed as frequency and percentage. Biochemical parameters were evaluated with the Shapiro-Wilk test and revealed that they did not show normal distribution (p<0.05). While comparing these variables to study groups, the Mann-Whitney U test or Kruskal Wallis tests were used. In order to determine the difference between groups, Dunn’s multiple comparison test was used. Additionally, Spearman correlation analysis was used to evaluate the association between numerical variables. Analyses were performed using SPSS 22.0, and p<0.05 was determined as the level of statistical significance.

## Results

We enrolled 112 patients with HF and 50 healthy controls. The mean age of the total study population was 59.57 ± 9.24 years. The baseline characteristics of the patients and controls were summarized in Table 1. Mean age was similar both between patient and control groups and between patients with different HF groups. As a marker of disease severity and prognosis, the hallmark marker of HF, N-terminal pro-B-type natriuretic peptide (NT-proBNP) median levels were significantly higher in the patient group compared to healthy controls (660 (291 -1000) vs. 92 (78 -102) p< 0.001). Also, there was a significant increase in NT-proBNP levels with decreasing ejection fraction (p<0.01). Serum GSK-3β levels were significantly increased in HF patients compared to healthy control (117.26 (45.39 -223.85) vs 13.91 (5.6 -23.3) p<0.001). HF patients were divided into three groups based on left ventricular ejection fraction (LVEF): a) HFpEF where LVEF is > 50%, b) HFmrEF where LVEF is > 40% but <50% and c) HFrEF where LVEF is <40%. While considering different HF groups based on LVEF, GSK-3β levels were lowest among patients with HFrEF (median 38.72) and highest among patients with HFpEF (median 236.44) (p<0.001). While evaluating patients with HF, in three groups based on LVEF, GSK-3β levels were significantly lower among patients with lower left ventricular ejection fractions. Among patients with HFpEF, median GSK-3β levels were 236.44, whereas serum GSK-3β levels were 129.2 for patients with HFmrEF and 38.72 for patients with HFrEF (p<0.001). Serum BNP levels were also significantly increased with decreasing ejection fractions (p<0.001). As a marker of inflammation and prognosis, hs-CRP levels and NLR ratios were similar between different HF groups based on LVEF. In-group differences and comparisons are summarized in the Central Figure and Table 2.

**Table 1:** – Baseline characteristics of the patients and controls

	Controls	Patients with heart failure	p
	Median (Q1-Q3)	Median (Q1-Q3)	
GSK-3β (ng/mL)	13.91 (5.6 -23.3)	117.26 (45.39 -223.85)	0.001***
NT-proBNP (pg/mL)	92 (78 -102 )	660 (291 -1000 )	0.001***
LVEF (%)	55 (55 -57 )	44.5 (35 -53.5 )	0.001***
hs-CRP (mg/L)	5 (2 -8.4)	4.78 (2 -7.05 )	0.405
Leukocyte	9 (7.21 -11.53)	9.71 (7.21 -11.53)	0.878
Platelet	258.5 (225 -311 )	281 (237 -333 )	0.252
Lymphocyte	2.23 (1.78 -4.87)	2.31 (1.5 -3.58)	0.187
Neutrophil	7.1 (4.96 -7.77 )	6.01 (4.44 -7.77)	0.330
Creatinine	0.87 (0.71 -1.01)	0.77 (0.7 -0.99)	0.148
eGFR	82.36 (65 -109 )	99.5 (72 -109.5 )	0.200
NLR	2.06 (1.12 -4.02)	2.32 (1.61 -4.01)	0.212
PLR	111.05 (57.7 -150.25)	123.93 (79.21 -180.99)	0.069

***p<0.001; Mann-Whitney U test. GSK-3β; glycogen synthase kinase 3 beta, NT-proBNP: N-terminal pro-brain natriuretic peptide; LVEF: left ventricular ejection fraction; hsCRP: highly sensitive C reactive protein; eGFR: estimated glomerular filtration rate (mL/min/1.73 m^2^); NLR: neutrophil to lymphocyte ratio; PLR: platelet to lymphocyte ratio.

**Table 2:** – In-group differences and comparisons

Variables	Healthy controls and types of heart failure	Median (Q1-Q3)	p
GSK-3β (ng/mL)	Healthy controls	13.91 (5.6 -23.3)	0.001***
	HFrEF	38.72 (23.15 -67.31)	
	HFmrEF	129.2 (78.9 -199.63)	
	HFpEF	236.44 (132.89 -432)	
NT-proBNP (pg/mL)	Healthy controls	92 (78 -102)	0.001***
	HFrEF	1110 (817.5 -2220)	
	HFmrEF	607 (430 -780)	
	HFpEF	225 (180 -312)	
hs-CRP (mg/L)	Healthy controls	5 (2 -8.4)	0.425
	HFrEF	5 (2.44 -9)	
	HFmrEF	5 (1 -6)	
	HFpEF	3.64 (2 -6)	
Leukocyte	Healthy controls	9 (7.21 -11.53)	0.998
	HFrEF	9.86 (7.12 -11.77)	
	HFmrEF	9.87 (7.21 -11.06)	
	HFpEF	8.9 (7.67 -12)	
Platelet	Healthy controls	258.5 (225 -311)	0.550
	HFrEF	272 (237.5 -358.5)	
	HFmrEF	280.5 (228 -321)	
	HFpEF	281 (238 -365)	
Lymphocyte	Healthy controls	2.23 (1.78 -4.87)	0.344
	HFrEF	2.31 (1.53 -4.87)	
	HFmrEF	2.31 (1.56 -2.75)	
	HFpEF	2.34 (1.42 -3.12)	
Neutrophil	Healthy controls	7.1 (4.96 -7.77)	0.025*
	HFrEF	7.59 (5.41 -8.33)	
	HFmrEF	5.95 (4.35 -6.87)	
	HFpEF	4.99 (4.37 -7.77)	
NLR	Healthy controls	2.06 (1.12 -4.02)	0.601
	HFrEF	2.13 (1.12 -4.62)	
	HFmrEF	2.62 (1.67 -3.95)	
	HFpEF	2.28 (1.87 -3.49)	
PLR	Healthy controls	111.05 (57.7 -150.25)	0.116
	HFrEF	116.22 (53.62 -175.07)	
	HFmrEF	119.25 (86.34 -161.04)	
	HFpEF	147.33 (101.08 -190)	

***: p<0.001 (statistically very strong significant difference). *: p<0.05 (statistically significant difference). GSK-3β; glycogen synthase kinase 3 beta; NT-proBNP: N-terminal pro-brain natriuretic peptide; LVEF: left ventricular ejection fraction; hsCRP: highly sensitive C reactive protein; eGFR: estimated glomerular filtration rate (mL/min/1.73 m^2^); NLR: neutrophil to lymphocyte ratio; PLR: platelet to lymphocyte ratio.

## Discussion

In this study, we evaluated established markers for prognosis among patients with HF, and as a new and promising marker, we measured serum GSK-3β levels and compared results to healthy controls. The main finding of the study was that serum GSK-3β levels had significantly increased among patients with HF. Also, with reducing ejection fraction, GSK-3β levels had a trend toward a reduction. The highest levels of GSK-3β levels were recorded among patients with HFpEF.

Glycogen synthase kinase (GSK) has two isoforms, α and β. For mechanisms specific to the heart, β isoform has gained much interest. Recent studies revealed that GSK-3β has an important role in ischemic preconditioning, postconditioning, hypertrophy, and HF. Among various pathways and mechanisms, the effect of GSK-3β on cardiac physiology is exerted by phosphorylation of transcriptional regulators and translational initiation factors.^8^ Activated GSK-3β prevents hypertrophic growth of myocytes, and especially in the process of advancing HF, GSK-3β is inhibited probably as a protective mechanism.^9^ In our study, serum GSK-3β levels were highest among patients with HFpEF. With reducing ejection fractions, GSK-3β levels were constantly decreasing, and this difference had reached a strongly significant difference (p<0.001). Our findings support previous research findings that, with advancing HF, GSK-3β is downregulated as a protective mechanism. Another support for this finding comes from an animal study. Kirk et al. evaluated dog hearts with HF with dyssynchrony.^1^º They used cardiac resynchronization therapy (CRT) for these animals and found that GSK-3β was deactivated in these hearts and reactivated by CRT. Also, CRT improved the calcium responsiveness of myofilaments through GSK-3 reactivation. This finding also suggests a possible therapeutic approach for HF therapies in the near future.

We measured serum BNP levels, which is accepted as the standard biomarker for HF both at the diagnosis and determination for prognosis and also evaluating the response to treatment.^2^ As compared to healthy controls, median NT-proBNP levels were significantly higher among patients with HF. This association was positively correlated with GSK-3β levels, which were also significantly increased among patients with HF compared to healthy controls. From this point, we can assume that GSK-3β may be used as a novel biomarker for HF like BNP. However, further studies with a greater number of patients are needed to clarify this association.

As an established risk factor for future cardiovascular and especially coronary events, we also measured serum high-sensitivity C-reactive protein (hsCRP) levels. Both for coronary artery disease and HF, hsCRP is an established marker for prognosis. Serum levels of hs-CRP were similar between healthy controls and patients with HF. Other widely studied markers of systemic inflammation are neutrophil-to-lymphocyte ratio (NLR) and platelets-to-lymphocyte ratio (PLR). Systemic inflammation is accepted as a key pathogenetic step both for the development of atherosclerosis and HF. Also, an increased level of systemic inflammation is accepted as a poor prognostic marker for acute coronary events and chronic HF. In their large meta-analysis, Dong et al. evaluated 9406 patients from eight studies and revealed that NLR is a poor prognostic marker for patients with recent acute coronary syndrome, and higher baseline NLR values were associated with increased in-hospital mortality.^11^ Uthamalingam et al. evaluated patients hospitalized for acute decompensated HF and revealed that increased NLR values were independently associated with long-term mortality.^12^ In our study, median values of NLR were similar both for healthy controls and for patients with HF (p:0.212). Also, median NLR values were similar across different HF subgroups (p:0.601). The relatively low number of patients can explain this lack of difference. Also, we did not enroll patients with acute decompensation. Since NLR is generally a marker of acute inflammation and hence acute decompensation of HF would probably cause in elevation of calculated NLR values.

### Study limitations and future perspectives

One limitation of our study was the relatively small number of patients enrolled. The higher number of patients would yield more clinically relevant and robust data. The glycogen synthase kinase beta pathway is gaining increased interest in the HF pathway. With an increased understanding of this pathway, new therapies for HF targeting the GSK pathway may become available in the near future.

## Conclusion

Serum levels of glycogen synthase kinase 3 beta (GSK-3β) is significantly increased among patients with HF compared to healthy controls. Also, GSK-3β levels showed a significant reduction with reduced ejection fraction, pointing towards a possible protective mechanism and also a therapeutic target for the treatment of HF.
